# An Investigation into the Role of Lifestyle Factors in SuperAgers in UK Biobank

**DOI:** 10.1002/alz70860_105062

**Published:** 2025-12-23

**Authors:** Philippa Watson, Megan Smith, Ivan Koychev, John Gallacher, Sarah Bauermeister

**Affiliations:** ^1^ University of Oxford, Oxford, Oxon, United Kingdom; ^2^ University of Oxford, Oxford, Oxfordshire, United Kingdom; ^3^ Imperial College London, London, London, United Kingdom

## Abstract

**Background:**

It is widely acknowledged that cognitive abilities tend to decline with age; however, there exists considerable intra‐individual variability in the rate of decline. While many individuals report varying degrees of cognitive decline, a small subset known as “SuperAgers” exhibits cognitive abilities comparable to those several decades younger. Our understanding of which factors enable SuperAgers to maintain their superior cognitive abilities is inconclusive. It is hypothesised, however, that the factors safeguarding against cognitive decline may also play a role in maintaining exceptional cognitive abilities.

**Methods:**

This study leverages data from the UK Biobank cohort. SuperAgers were defined as those over 65 years old at baseline who scored above the mean of 37‐45 year olds on a test of numerical memory performance at baseline and who performed within one standard deviation of the mean for fluid intelligence at baseline. Structural equation modelling will be employed to conduct a multivariate statistical analysis investigating the association between engagement in various lifestyle factors (e.g., mental health, physical activity, social engagement, and biomedical factors) in SuperAgers and cognitively normal agers (CN's).

**Results:**

The study comprised 19,557 participants (4,261 females), with 13,088 meeting the criteria for SuperAgers and 6,469 for CN's. SuperAgers exhibited significantly lower levels of physical activity engagement (*t* = 7.56, *p* < .001), were more likely to have been ever smokers (*χ* ^2^ = 188.45, *p* = .003), drank more units per week (*t* = ‐17.76, *p* < .001), had worse adherence to the MIND diet (*t* = 6.12, *p* < .001), had higher levels of social visits (*t* = ‐9.72, *p* < .001), and had lower levels of anxiety (*t* = 11.40, *p* < .001) and depression (*t* = 9.32, *p* < .001). No significant differences were observed between SuperAgers and CN's regarding BMI levels, hypertension rates, total medications, social activity engagement and healthy sleep scores (*p* > .05). Additional analyses are ongoing and will be completed by the conference date.

**Conclusions:**

The findings from this study will provide valuable insights into the contribution and significance of various modifiable lifestyle factors in preserving cognitive function during older age.

